# The gravity dependence of pharmacodynamics: the integration of lidocaine into membranes in microgravity

**DOI:** 10.1038/s41526-019-0064-5

**Published:** 2019-03-06

**Authors:** Florian P. M. Kohn, Jens Hauslage

**Affiliations:** 1 Department of Membrane Physiology (230b), Institute of Physiology, University of Hohenheim, Stuttgart, Germany; 2 German Aerospace Center (DLR), Institute of Aerospace Medicine, Gravitational Biology, Linder Hoehe Cologne, Germany

## Abstract

To realize long-term manned space missions, e.g. to Mars, some important questions about pharmacology under conditions of different gravity will have to be answered to ensure safe usage of pharmaceuticals. Experiments on the International Space Station showed that the pharmacokinetics of drugs are changed in microgravity. On Earth, it is well known that the incorporation of substances into cellular membranes depends on membrane fluidity, therefore the finding that membrane fluidity is gravity dependent possibly has effects on pharmacodynamics of hydrophobic and amphiphilic substances in microgravity. To validate a possible effect of gravity on pharmacodynamics, experiments have been carried out to investigate the incorporation of lidocaine into plain lipid membranes under microgravity conditions. In microgravity, the induced increase in membrane fluidity associated with lidocaine incorporation is smaller compared to 1*g* controls. This experiment concerning the gravity dependence of pharmacodynamics in real microgravity clearly shows that the incorporation of amphipathic drugs into membranes is changed in microgravity. This might have significant impact on the pharmacology of drugs during long-term space missions and has to be investigated in more detail to be able to assess possible risks.

## Introduction

Long-lasting manned space missions beyond Earth's orbit, e.g., to Mars are already planned by space agencies and private corporations.^1^ Then humans will have to live in closed environments under space conditions including microgravity for long periods without immediate access to medical or pharmacological support from Earth. To minimize the risk for astronauts, the effects of relevant drugs under space conditions will have to be investigated in detail before such manned mission will start. To assess whether the action of pharmaceuticals is different in weightlessness compared to Earth, pharmacokinetics (PKs) and pharmacodynamics (PDs) have to be investigated in more detail to establish a “space pharmacology”.^2,3^ According to National Aeronautics and Space Administration, there is evidence for inadequate treatment of astronauts during and after a mission.^2^

Limited data are available for PKs in real weightlessness, showing that, e.g., the peak concentration of acetaminophen in the saliva is significantly smaller, and the time to reach it is significantly longer compared to Earth.^4,5^ Unfortunately, no data exist for PDs in real weightlessness yet. Up to now, only ground-based experiments (“simulated microgravity”), e.g., from bed rest studies^6^ have been performed in 1*g*. Lidocaine is a local anesthetic and antiarrhythmic drug included in the International Space Station (ISS) pharmacy, with potential variability in its PKs due to metabolism by polymorphic enzymes.^7^ For this drug, PK changes have been detected in subjects exposed to 4-day head down tilt tests.^8^

On Earth, it is well known that the incorporation of hydrophobic, amphiphilic, and other similar drugs into membranes depends on membrane fluidity,^9^ as well as it has been shown that the incorporation of Alamethicin into lipid monolayers is surface pressure dependent.^10^ Furthermore, it was demonstrated that ligand–receptor interactions are membrane fluidity dependent, using the nicotinic acetylcholine receptor as a model system.^11^

From experiments in clinorotation^12^ and in real microgravity and hypergravity^13,14^ it is known that membrane fluidity itself is affected by gravity. In microgravity membrane fluidity is increased; in hypergravity it is decreased.^13^

Following that the incorporation of hydrophobic (and other) substances and ligand–receptor interactions depends on membrane fluidity, which in turn is gravity dependent, it can be concluded that a major part of pharmacological relevant substances might have changed PDs in microgravity compared to 1*g* conditions.

A major class of drugs in the above stated field of space pharmacology are anesthetics, which partially act via membrane incorporation.^15^ They are of special interest as a change in their PD properties could have severe consequences for astronauts.

To assess whether PDs of hydrophobic anesthetics is indeed affected by gravity, we have used lidocaine as a model substance, as it is well known that it incorporates into the membrane,^16^ increasing membrane fluidity.^17^ The lidocaine-induced fluidization of membranes was measured by fluorescence polarization (FP) in 1*g* laboratory conditions and in the microgravity phase during a sounding rocket flight.

## Results

During the microgravity phase of a rocket flight, the incorporation of 16 mM lidocaine into plain lipid vesicle membranes was measured by FP as described in the Methods section.

**Fig. 1:**
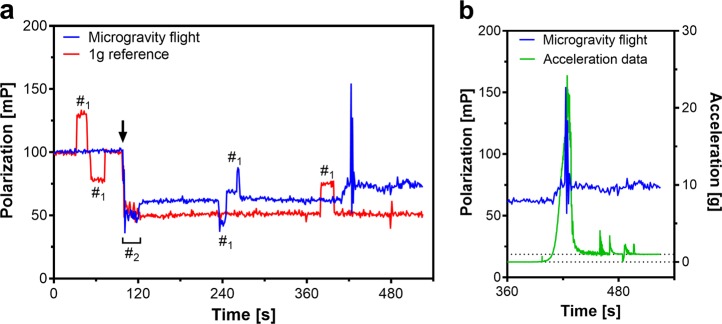
**a** Normalized data of the changes in fluorescence polarization (FP) of the microgravity experiment and 1*g* ground reference. In both cases, upon addition of 16 mM lidocaine (start of mixing is indicated by arrow; mixing indicated by #_2_; artifacts caused by moving air bubbles are marked by #_1_), FP decreased. A reduced FP indicates a decrease in membrane viscosity or an increase in membrane fluidity. It is visible that, during microgravity, the increase membrane fluidity is less compared to 1*g*. The big spike was caused by the high *g*-load (24.2*g*) during the deployment of parachute. One datapoint (second 428) during the deployment was removed as an artifact due to the mechanical shock. **b** FP flight data compared to the acceleration profile (vector addition of all three axes *x*, *y*, and *z*). At the end of the flight, the average gravity increases again, the big spike indicates the opening of the parachute. With onset of gravity, FP slightly increased again. The dotted lines indicate 0*g* and 1*g*

The data from the microgravity experiment was compared with the 1*g* ground reference experiment. In both experiments, 16 mM lidocaine decreased FP, which means that the fluidity of the membrane is increased (Fig. 1a). Compared to the 1*g* reference, the lidocaine-induced increase in membrane fluidity was smaller in microgravity.

After the deployment of the parachute (big spike in Fig. 1a), the FP slightly increased, which means that membrane fluidity was decreased. This was due to the increased gravity conditions caused by the reentry into the atmosphere and the deployment of the parachute (Fig. 1b).

**Fig. 2:**
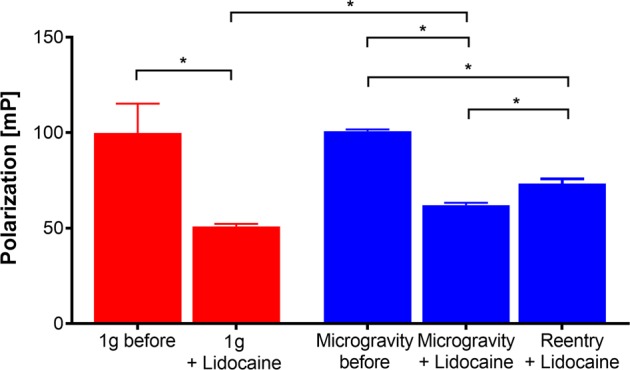
Statistical analysis of the data from the flight and 1*g* experiment. In 1*g* and in microgravity, 16 mM lidocaine significantly reduced fluorescence polarization (FP), so membrane fluidity is increased. A comparison between the lidocaine-induced changes of FP reveals a significant smaller decrease in microgravity compared to 1*g*. During the reentry phase, FP significantly increases again due to the general gravity dependence of membrane fluidity itself, but it does not recover to the values before the addition of lidocaine as this decreases FP. *n* (from left to right) = 98, 287, 98, 287, 94. Mean ± SD, Welch–analysis of variance and Games–Howell post hoc test. *p < .0005

A statistical analysis of the flight and 1*g* data (Fig. 2) revealed that the increase in membrane fluidity of asolectin vesicles after the addition of 16 mM lidocaine is significant, but more importantly, it showed that the increase was significantly less in microgravity compared to 1*g*. After the onset of gravity, membrane fluidity was significantly decreased compared to microgravity conditions.

**Fig. 3:**
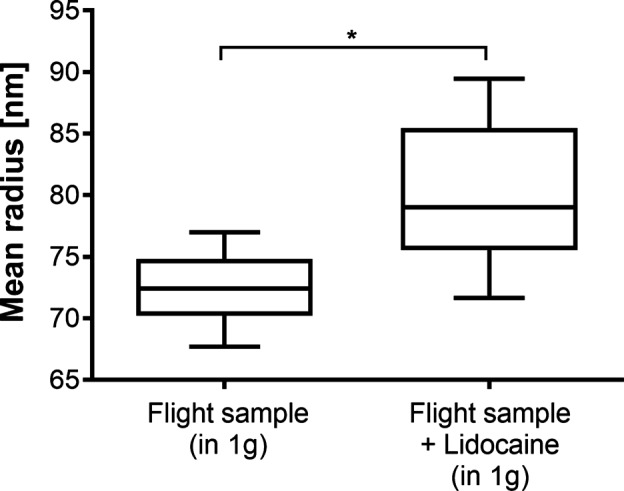
The change in vesicle size after the addition of 16 mM lidocaine measured in 1*g* after the flight. The untreated vesicle preparation of the rocket mission (not flown) was compared to the returned flight sample. The mean vesicle radius increased from 72.51 nm before the addition to 80.18 nm afterwards. *n* = 20. Boxplot: median, 25 and 75 percentile, whiskers min to max. Unpaired *t* test, *p < .0001

To assess whether lidocaine incorporated into the lipid bilayer, an additional 1*g* experiment was executed after the rocket flight. The vesicle size of the untreated vesicles (not flown but same vesicle preparation) and of the returned flight sample was measured by dynamic light scattering (DLS) in the laboratory. The vesicle size of the returned flight sample (treated with 16 mM lidocaine during the flight; no additional treatment on Earth) increased by 10.6%. No time-dependent variation of vesicle size was observed (Fig. 3).

## Discussion

There is experimental evidence that lidocaine affects neuronal sodium channels directly by binding to the D4-S6 region of the α-subunit^18^ and indirectly by modification of membrane properties.^19,20^ How and to what extent these direct and indirect effects on ion channels are intertwined for the pharmacological effect is still under discussion.^15,17,21^

The direct effect is preferred by many researchers, but it is well known that ion channel parameters, as the closed-state probability of the nicotinic acetylcholine receptor, for example, clearly depend on membrane fluidity.^11^ Dissolved lidocaine exist in uncharged and positively charged form,^22^ the uncharged molecules can diffuse through the membrane and interact with ion channels directly,^17^ and the charged lidocaine accumulates in the hydrophobic part of the lipid membrane.^16^

By incorporation into the membrane, lidocaine increases membrane fluidity^23,24^ and decreases membrane thickness.^25^ This can be measured by optical methods as FP,^23,26,27^ for instance. However, no absolute values of membrane viscosity can be obtained with this technique. Nevertheless, it can be used for relative assessments, e.g., if the fluidity of a sample decreases or increases or if ≥2 samples differ after treatment with different lidocaine concentrations.

With these prerequisites, the presented experiment now clearly shows two things. First, the incorporation of lidocaine into the membrane increases membrane fluidity (FP is decreased). The increase in vesicle size after the addition of lidocaine (Fig. 3) is a strong support that lidocaine molecules really incorporate into the membrane. More molecules per membrane area could correlate with an increase in vesicle size.

There are also light scatter experiments indicating that the size of spheroid cells is increased in microgravity.^28^ As a lidocaine-induced decrease in membrane thickness was found,^25^ an interesting experiment would be to monitor membrane thickness in microgravity and hypergravity to evaluate whether this has an effect on the found general gravity dependence of membrane fluidity.^13^

Second and more importantly, the lidocaine-induced increase in membrane fluidity is gravity dependent. Compared to the 1*g* ground control, the increase in fluidity is significantly lower in microgravity.

The demonstrated decrease in membrane fluidity (indicated by the increase of FP) after the opening of the parachute and the consequential onset of gravity (Fig. 1a) was expected, as an increase in gravity should decrease membrane fluidity.^13^ However, as there is a lidocaine-induced increase in fluidity, the FP signal does not fully recover to the measured value before the mixing. Nevertheless, the general gravity dependence of membrane fluidity^13,14^ could be verified. After the initial 24.2*g* shock during parachute deployment, the average *g*-load was ≥1*g*. The fast high-*g* spikes are not visible in the FP signal, but this is due to the limit in sampling rate of the system.

From these findings, it is evident that the PD properties of lidocaine are different in weightlessness compared to 1*g* on Earth. A general gravity dependence of PDs was postulated before,^13^ and the few experiments on board the ISS focusing on PDs^2,4^ strongly suggested that PDs should also be affected by gravity, but it was never investigated up to now.^2,3^

The presented sounding rocket experiment is an experiment in real microgravity, which shows that at least the PDs of hydrophobic and amphiphilic substances indeed is gravity dependent. Up to now, only experiments under so called “simulated gravity” (e.g., bed rest studies) exist.^6^ These experiments can be used to monitor general physiological adaptations due to changes in, e.g., blood distributions and to investigate microgravity-related effects, but as they are still executed in 1*g* conditions, these types of experiments cannot simulate changes in membrane fluidity, as this is only changed in real microgravity and hypergravity.^13,29^ Therefore, experiments that investigate the molecular and biophysical principles of membrane interaction (which are parts of PDs) or use hydrophobic substances have to be conducted in real microgravity (and hypergravity).

It is known that the properties of the nicotinic acetylcholine receptor are affected by the biophysical properties of the membrane.^11^ Following this, it can be postulated that also receptor–ligand interactions and other signal cascades should be affected by gravity^14^ as most of the receptor proteins are membrane proteins. From experiments during parabolic flight missions (generating real microgravity and hypergravity), electrophysiological data are available, indicating that synaptic transmission at the motor end plate is also affected.^30^ Of course, it has to be investigated in more detail to be able to make a definitive statement.

Coming back to the ascertained gravity dependence of lidocaine PDs, the question arises about the mechanism. One plausible explanation is that the number of incorporated molecules is reduced in microgravity due to the general gravity dependence of membrane fluidity. From Earth-bound experiments, we know that the increase in membrane fluidity depends on lidocaine concentration. It is increased with rising lidocaine concentration.^31^

If the result of this experiment is interpreted to state that less lidocaine molecules are integrated into the membrane, this would have a huge impact on space pharmacology. It would mean that in microgravity the dose of hydrophobic medication (as many anesthetics are) must be increased. This could—in worst case—negatively influence the therapeutic index. The same would be true for many other drugs as outlined already shortly. Of course, this has to be verified with additional experiments not only on the pharmacokinetic but also on the pharmacodynamic level as well as with efficacy studies.

Consequently, on long-lasting human space missions, this has to be taken into account, and sufficient data have to be provided by setting up a research field of space pharmacology,^2^ which not only focuses on PKs, where first results already have been delivered,^2,32,33^ but also has to include PDs as this part was neglected until now. Data from PK and PD experiments have to be integrated for better understanding the gravity dependence of the effect of pharmaceuticals to assess the yet unknown risk for astronauts. As PD experiments can be conducted with artificial systems and cell cultures, these experiments can easily be repeated with a high number of samples, delivering good statistical data. Therefore, data from these basic experiments can support pharmacological experiments with human test subjects in space, as these experiments usually are conducted with a limited number of subjects, as the pool of possible test subject is limited to (mainly) astronauts and volunteers during parabolic flights.

In addition, basic research concerning the biophysical and molecular principles behind drug–target interactions can be used to optimize experiment design and analysis of PK experiments with astronauts. By revealing that the biophysical membrane properties are affected by gravity^13^ and that this affects the incorporation of anesthetics, as shown in this manuscript, the statement that “*the intrinsic ability of a drug to cross a membrane or to be actively transported is unlikely to be changed in microgravity*”^32^ could be disproved.

A recent review from 2017 revealed that this topic is also of high interest for jet fighter pilots, as a risk for a difference disposition of chemicals in the body during high-*g* maneuvers was found,^34^ but—similar to astronauts in weightlessness—the risk cannot be assessed yet as there is not enough data.

Therefore, to further improve the knowledge about the interaction of PDs and gravity, a systematic investigation of membrane interactions and receptor–ligand interactions of relevant pharmaceuticals and substances is necessary, not only in microgravity but also in hypergravity.

With an optimized hardware, other, easier accessible research platforms as parabolic flights or drop towers can be used. The used hardware is based on a so called stopped-flow system, which is regularly used to investigate PD reactions. Depending on the mixing speed, extremely fast reactions can be measured (in the range of milliseconds for industrial systems). As it was shown in the data, the lidocaine-induced change in membrane fluidity is within the range of seconds and reaches a stable plateau. A fast system with a faster mixing time could be used in parabolic flights or drop tower to significantly increase the amount of available data. The number of experiments could also be increased by performing parallel experiments during the same flight. Then also the different gravity phases can be investigated in more detail by, e.g., starting control experiments in 1*g* to monitor the general gravity dependence of membrane fluidity throughout the whole flight. Also, centrifuges can be used to investigate the gravity dependence of PD in increased gravity.

As vesicle preparations are stable up to several weeks, this type of experiment could be a good candidate for an automated ISS experiment, as no cell culture system is needed.

## Methods

### Sounding rocket

The experiment was part of the Mapheus program of the German Aerospace Center (DLR). It was carried out during the Mapheus 6 flight at Esrange Space Center, Sweden. The rocket was launched on 14 May 2017.

This sounding rocket system was an unguided two-stage rocket and consisted of a VSB-30 vehicle^35^ and the scientific payload on top. During the flight, a microgravity time of up to 6 min can be achieved with a microgravity quality of about 10^−3^–10^−4^*g*. The total flight time of Mapheus 6 (from launch to landing of the payload) was 858 s.

### Vesicle preparation

The vesicles were made from asolectin (from soybean) as the preparation of these vesicles is easy and can be established in a laboratory quite fast. Asolectin is a mixture of unsaturated phospholipids whose main components are soybean phosphatidylcholine (25%), phosphatidylethanolamine, and phosphatidylinositol.^36^ It is regularly used in pharmacological experiments^37^ as it is easy to obtain lipid extract of a biological membrane.

Asolectin was dissolved in chloroform (10 mg/ml). Afterwards 1 ml of this solution was filled in a round-bottomed flask and the solvent was removed in a rotary evaporator (300 mbar, 1 h) until the lipid remained as a dry thin film. To remove the last residue of solvent, the flask was stored in a fume hood overnight. After these steps, the lipid film was rehydrated with 20 ml of phosphate buffered saline (PBS; without Ca^2+^ and Mg^2+^) and the fluorescent dye DPH (1,6-diphenyl-1,3,5-hexatriene; dissolved in dimethyl sulfoxide (DMSO); end concentration 10 µM) for 1 h at 30 °C. To protect the dye from light, the flask was completely covered with aluminum foil. The rehydrated lipid was sonicated (1 h; temperature was kept below 40 °C) and afterwards centrifuged for (30 min; 3000 × *g*; room temperature). The supernatant containing the DPH-doped vesicles was carefully removed and stored in the fridge in an opaque tube (4 °C). The dyed vesicles were stable for 14 days. Nevertheless, during the sounding rocket mission, fresh vesicles were made 24 h before each launch attempt.

### Lidocaine solution

Lidocaine hydrochloride was dissolved in PBS (without Ca^2+^ and Mg^2+^). A stock solution of 32 mM was used to obtain a final concentration of 16 mM after mixing it with the asolectin vesicle solution. This concentration was determined in previous calibration experiments during the design of the experiment hardware.

### Measurement of membrane fluidity

Changes in membrane fluidity were measured by *t*-format FP. In short, a fluorescent probe, DPH in this experiment, was excited by polarized light. The emission wavelength was measured by two photomultipliers (PMT), again with polarizers parallel (0°) and perpendicular (90°) to the excitation light.^38^ For this experiment, an excitation wavelength of 365 nm was used. The PMTs recorded emission wavelengths >430 nm.

With these two fluorescence intensities, the rotational speed of the fluorophore can be measured and this can be used to investigate membrane fluidity^26^ and is used to measure the effect of anesthetics such as lidocaine on membrane fluidity.^17,23^ The fluorescence signals of the two PMTs were used to calculate FP in mP.^39^ As the polarization signals are sensitive to the cuvette position, the signals of the experiments were normalized to 100 mP to measure the relative changes in fluidity in 1*g* and microgravity.

### Measurement of vesicle size

The size of the flight sample (before and after addition of 16 mM lidocaine in flight) was measured by DLS after landing of the experiment. Twenty separate measurements have been made for each condition, with 50 µl of sample per measurement.

#### 1*g* reference experiment

The ground reference experiment was performed on the same day as the microgravity experiment in the recovered experiment hardware. Ambient conditions (pressure and temperature) were identical to the flight conditions and the same vesicle and asolectin preparation as used for the rocket mission was used to ensure identical experiment conditions.

### Experiment hardware

**Fig. 4:**
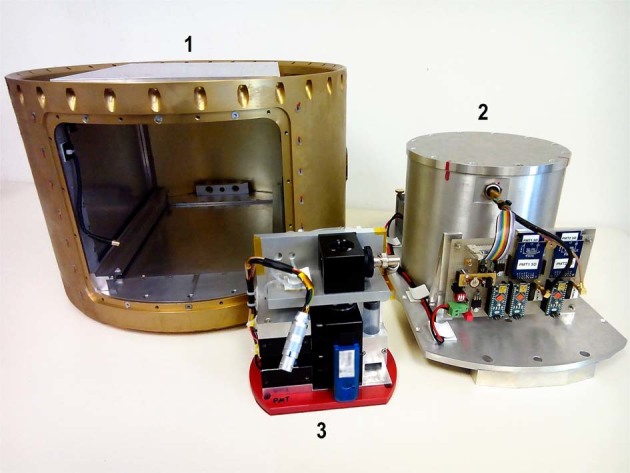
Picture of the experiment hardware. (1) Rocket structure, (2) late access unit (LAU), and (3) experiment module. The experiment module fits into the pressure chamber of the LAU. The LAU is integrated into the structure housing shortly before launch

The experiment hardware was designed and built to be usable during a sounding rocket flight, and all required tests (e.g., on *g*-load and vibrations) were passed successfully. The hardware consists of three units: (1) the rocket structure, (2) the late access unit (LAU), and (3) the experiment module (Fig. 4).

### Experiment module

The experiment module contained the fluidic and the optical system. Two standard syringes (Luer taper; 10 ml) were operated by a motor-driven syringe pump. They were connected to a coin-shaped quartz glass cuvette (volume approximately 50 µl) by silicone tubes. The content of both syringes was mixed in a t-connector that was connected to the inlet port of the cuvette. To avoid overpressure in the system, a waste container was connected to the outlet port of the cuvette.

**Fig. 5:**
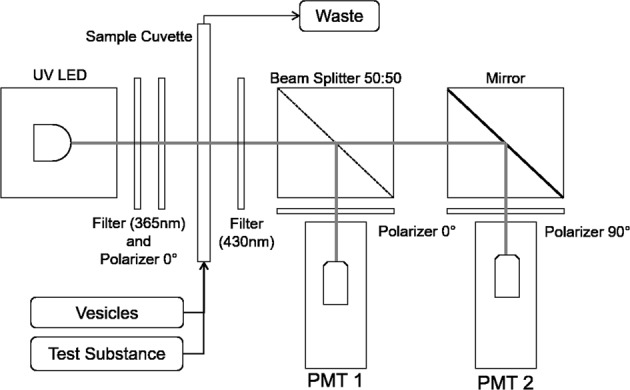
Schematic of the experiment optical set-up and fluidic system. Vesicles and test substances are mixed and transferred to the sample cuvette. Fluorescence polarization is measured continuously for 525 s

Fluorescence was excited by a ultraviolet light-emitting diode (UV-LED) with an additional bandpass filter (365 nm ± 10 nm). By using a long pass filter (430 nm), the excitation wavelength was shielded from the two digital PMTs (Hamamatsu H7155) and thus only emission wavelength was recorded. FP was measured by using three polarizers: one in front of the UV-LED, and one in front of each PMT (parallel and perpendicular to the excitation polarizer). Before each experiment, the experiment unit was placed inside the pressure-tight chamber of the LAU and was connected to the power and control system. A basic schematic of the fluidic and the optical system is shown in Fig. 5.

### Late access unit

The LAU had three separate functions. (1) A vacuum-tight chamber allowed experiments to be carried out under normal pressure (approximately 1000 mbar) during the flight of the rocket. (2) It served as power supply for the complete experiment. It was powered by accumulators (12 V, 4800 mAh, NiMH), which were located outside the vacuum chamber. (3) The electronics to control the experiment and to handle the data were located on the aluminum base plate in front of vacuum chamber. Via a vacuum tight plug, data and power lines were connected to the experiment unit inside the chamber.

The fluorescence signal from each PMT was handled by a dedicated microcontroller (Arduino Nano 3.0) and was stored on two memory cards. A third Arduino served as a master controller. It was connected to the main rocket computer and operated the complete experiment after the proper start of experiment (SOE) signal was given by the rocket system. It also synchronized the real-time clock and controlled the subordinate Arduinos and the experiment module (pumping and switching of the UV-LED).

### Ambient data

To be able to execute the 1*g* reference experiments with the same temperature profile as given during the rocket flight, temperature was continuously measured at two locations inside the pressure chamber of the LAU (directly at the cuvette and near the inner side of the wall) by two battery-powered data loggers (Arexx BS-30).

### Experiment procedures

Three hours before liftoff, two syringes were filled with (1) 3.5 ml of asolectin vesicle preparation and (2) 3.5 ml 32 mM lidocaine solution. The silicone tubes and the quartz cuvette were pre-filled with the same vesicle preparation to measure the FP baseline signal of the vesicle preparation without lidocaine after the experiment was started and to remove air bubbles from the system. After these steps, the experiment unit was mounted into the pressure chamber of the LAU and it was tightly sealed.

Two hours before the end of the countdown, the LAU was integrated into the rocket structure, the experiment was connected to rocket service module, and the experiment was set to standby.

With the onset of microgravity (approximately 70 s after liftoff), the SOE signal was given and the experiment was switched to active: First, the baseline FP of the unmodified DPH-doped asolectin vesicles was measured for 100 s. Subsequently, the syringe pump was activated and the content of the two syringes were mixed and the mixture was pumped into the sample cuvette (18-s pump duration; final lidocaine concentration 16 mM). Excess liquid was pumped into the waste syringe. The FP measurement was continued for additional 425 s to include the gravitational pull after reentry into the atmosphere and opening of the parachute.

### Materials

Asolectin, lidocaine hydrochloride, DPH, DMSO, and chloroform were purchased from Sigma-Aldrich, Germany. PBS (without Ca^2+^ and Mg^2+^) was purchased from Merck-Biochrom (Berlin, Germany).

The polarizers and optical filters were purchased from Edmund Optics Germany. The PMTs are from Hamamatsu Photonics (Hamamatsu, Japan). The electronics were purchased from R+S components Germany and Reichelt Elektronik (Sande, Germany). The metal components and the cuvette were manufactured at the workshops of DLR (Cologne, Germany) and the University of Hohenheim (Stuttgart, Germany).

### Data and statistical analysis

Major goal of this pathfinder experiment was to assess the question whether the incorporation of hydrophobic and amphiphilic substances is gravity dependent as previously proposed.^13^

Owing to volume and mass limitations of a sounding rocket mission, the flight data represent only one replicate. Statistical analysis was performed using SPSS 25 and GraphPad Prism 6. Data are expressed as mean ± SD of “*n*” of the 525 time points of the complete experiment. The mean of each group was calculated with a minimum of 94 time points (number of time points: 1*g* before: 98; 1*g*+lidocaine: 287; 0*g* before: 98; 0*g* lidocaine: 287; reentry+lidocaine: 94; presented in Fig. 2). Data were tested for normality using D’Agostino–Pearson omnibus normality test. Normally distributed data were analyzed for statistical significance using two-tailed Student's *t* test For non-normally distributed data, statistical significance among the different experiment conditions was assessed by Welch–analysis of variance and Games–Howell post hoc test.

### Reporting summary

Further information on experimental design is available in the Nature Research Reporting Summary linked to this article.

## Supplementary information

Reporting Summary

## Data Availability

The data that support the findings of this study are available from the corresponding author.

## Supplementary information

**Supplementary information** accompanies the paper on the *npj Microgravity* website (10.1038/s41526-019-0064-5).
